# Immunomodulatory potential of *Alternaria* mycotoxins on immune and intestinal cells: a comparative systematic in vitro study

**DOI:** 10.1007/s00204-026-04346-7

**Published:** 2026-04-28

**Authors:** Vanessa Partsch, Amina Selimagić, Francesco Crudo, Doris Marko

**Affiliations:** 1 Department of Food Chemistry and Toxicology, Faculty of Chemistry, University of Vienna, Währinger Str. 38, 1090 Vienna, Austria; 2 Doctoral School in Chemistry, Faculty of Chemistry, University of Vienna, 1090 Vienna, Austria

**Keywords:** *Alternaria alternata*, Food contaminants, Immunosuppression, NF-κB pathway, Innate immunity

## Abstract

*Alternaria* species produce structurally diverse mycotoxins that frequently contaminate food and are discussed in association with adverse health effects. However, systematic knowledge of their immunosuppressive potential is limited. This study evaluated seven *Alternaria* mycotoxins-alternariol (AOH), alternariol monomethyl ether (AME), altenuene (ALT), alterperylenol (ALTP), altertoxin I (ATX-I), altersetin (AST), and tentoxin (TEN)-in immune and intestinal cells. NF-κB reporter gene assays were performed in THP-1 monocytes and phorbol 12-myristate 13-acetate-differentiated macrophages (0.01-25 µM), while cytokine transcription and secretion (IL-6, IL-8, IL-10, TNF-α) were analyzed in Caco-2 and HCEC-1CT cells (0.1–30 µM) applying qRT-PCR and ELISA. All mycotoxins suppressed NF-κB activation in immune cells, though with differing potency. AOH, ALTP, and ATX-I were active at concentrations ≥ 1 µM in both models. AME and AST were more potent in macrophages (≥ 0.01 µM) than monocytes (≥ 5 µM). TEN suppressed the pathway at ≥ 0.1 µM in macrophages and ≥ 5 µM in monocytes, while ALT at ≥ 5 µM in both cell types. In intestinal models, AOH, AME, ALTP, ATX-I, and TEN modulated cytokine transcription, with stronger responses in non-tumorigenic HCEC-1CT cells. ALTP emerged as the most potent immunosuppressive compound, reducing IL-6, IL-8, and/or TNF-α secretion at concentrations ≥ 0.1 µM. AOH showed dual activity, suppressing IL-6/IL-8 at 30 µM, while modulating TNF-α in a cell-type-dependent manner (increased secretion in Caco-2, decreased in HCEC-1CT). This comparative study reveals immunosuppressive profiles of *Alternaria* mycotoxins in immune and epithelial cells, highlighting their relevance as food contaminants capable of modulating both intestinal and systemic immune responses.

**Supplementary Information:**

The online version contains supplementary material available at 10.1007/s00204-026-04346-7.

## Introduction

The immune system plays a critical role in protecting the organism from a wide array of environmental threats, such as pathogens and food-associated xenobiotics. As a complex network organized into specialized tissues, cells and messenger molecules, it is finely tuned to react adequately to external stimuli. While a fast and efficient reaction to danger signals is pivotal, deregulated inflammatory responses can lead to tissue damage and acute or chronic immune diseases (Marshall et al. 2018). The nuclear factor kappa-light-chain-enhancer of activated B cells (NF-κB) signaling pathway is one of the central mediators of inflammatory responses of the innate and adaptive immune system. The activation of the pathway leads to the transcription of numerous genes, including those encoding pro-inflammatory cytokines like interleukin (IL)-6, IL-8 and tumor necrosis factor-alpha (TNF-α), and is therefore essential for immunoregulation (Liu et al. 2017).

**Fig. 1:**
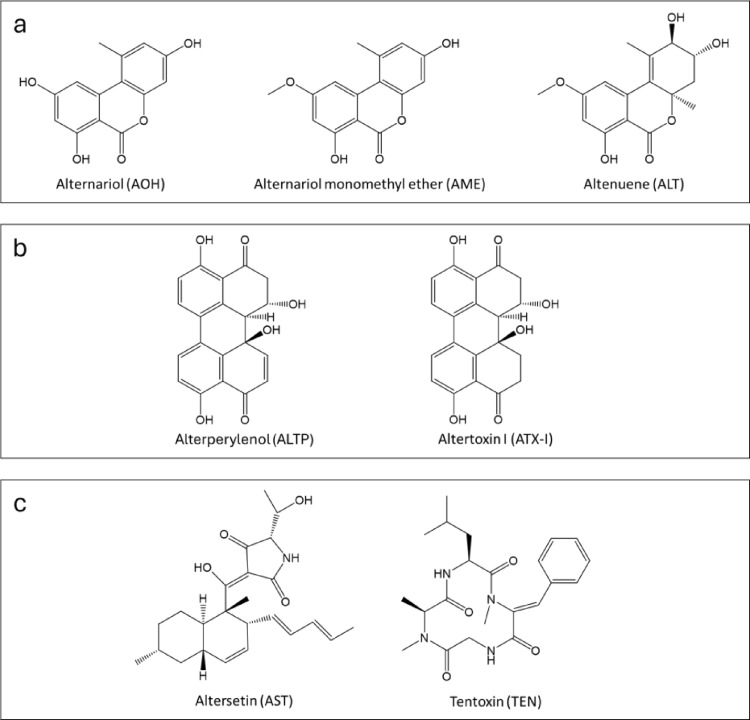
Chemical structures of the seven *Alternaria* mycotoxins investigated in this study. **a** shows dibenzo-α-pyrones, **b** perylene quinones, and **c** mycotoxins with miscellaneous structures

Among environmental contaminants, mycotoxins produced by *Alternaria* species are increasingly recognized for their potential to cause diverse adverse effects. These filamentous fungi frequently colonize a wide variety of raw materials and crops, such as cereals, fruits, vegetables, as well as the respective food products, thus causing widespread human exposure (Aichinger et al. 2021). Furthermore, they can produce an array of structurally diverse toxins, including over 70 chemically identified compounds. Based on their chemical structures, *Alternaria* mycotoxins can be sub-categorized into different groups. Accordingly, alternariol (AOH), alternariol monomethyl ether (AME) and altenuene (ALT) belong to the dibenzo-α-pyrones, alterperylenol (ALTP) and altertoxin I (ATX-I) to the perylene quinones, and altersetin (AST) and tentoxin (TEN) are categorized as *Alternaria* mycotoxins with miscellaneous structures (Fig. 1) (Louro et al. 2024; Aichinger et al. 2021; Gruber-Dorninger et al. 2017). While some *Alternaria* mycotoxins like AOH and AME have been shown to possess genotoxic and estrogenic properties (Lehmann et al. 2006; Hessel-Pras et al. 2019; Pfeiffer et al. 2007; Dellafiora et al. 2018), recent studies have also investigated their immunomodulatory potential. Several toxins, including AOH, ALTP, ATX-I and AST were shown to inhibit the lipopolysaccharide (LPS) induced activation of the NF-κB signaling pathway in THP-1 monocytes (Crudo et al. 2025; Partsch et al. 2025). Moreover, AOH and AME were found to decrease the secretion of IL-6 in LPS-stimulated RAW264.7 mouse macrophages (Grover and Lawrence 2017). AOH was also reported to downregulate protein levels of IL-6, IL-8 and TNF-α, while upregulating the secretion of the immunosuppressive cytokine IL-10 in THP-1 macrophages (Kollarova et al. 2018; Solhaug et al. 2016). Nevertheless, a systematic and comparative analysis of structurally distinct *Alternaria* mycotoxins regarding their capacity to interfere with inflammatory signaling is still lacking.

Monocytes and macrophages are key effector cells of the innate immune system and take over distinct roles in pathogen recognition, phagocytosis and cytokine signaling. While the circulating monocytes can rapidly respond to inflammatory stimuli by producing cytokines and chemokines, they can also migrate to the tissues and differentiate into macrophages, which act as antigen presenting cells, perform phagocytosis, and contribute to tissue repair. Among others, this process leads to altered surface marker expression and enhanced inflammatory responsiveness. Of note, the in vitro differentiation of monocytic THP-1 cells into macrophage-like cells (M0 macrophages) can be achieved by stimulation with phorbol 12-myristate 13-acetate (PMA) (Forrester et al. 2018). The highly dynamic differentiation process, which is amongst others under the control of the NF-κB pathway, results in two general phenotypes, the pro-inflammatory M1 and anti-inflammatory M2 macrophages (Wang et al. 2014). The different macrophage phenotypes can be distinguished by their surface marker expression profiles, which may also be modulated upon exposure to mycotoxins. In this context, co-incubation with AOH was reported to lead to a downregulation of the M0-associated surface markers CD14 and CD11b, while the monocytic marker CD71 was upregulated in PMA-differentiated macrophages. Thus, it was postulated that AOH interferes with the macrophage differentiation process, ultimately leading to immunosuppression (Solhaug et al. 2016).

Since ingestion is the primary route of exposure to mycotoxins, the gastrointestinal (GI) tract is one of the main targets of their toxicity (Maresca and Fantini 2010). In this context, the intestine serves not only as the principal site of nutrient absorption, but also as a dynamic immunological interface constantly exposed to dietary antigens, microbial metabolites, as well as foodborne mycotoxins (Bouhet and Oswald 2005). The intestinal barrier is formed by a monolayer of intestinal epithelial cells (IECs), which are covered by mucus, and represent the first cellular barrier encountered by ingested compounds. Beyond their classical barrier function, IECs actively take part in the body’s defense system by detecting microbial and inflammatory stimuli and by secreting messenger molecules, thus playing an important role in the coordination of local immune responses (Sarkar and Mitra 2022). Disruption of IEC function by mycotoxins may impair intestinal integrity, weaken immune defense mechanisms, and contribute to chronic inflammation or increased susceptibility to disease (Yao et al. 2024). Recently, it has been shown that in IL-1β-stimulated Caco-2 cells AOH downregulates IL-6, IL-8, and IL-1β transcription as well as IL-8 secretion (Schmutz et al. 2019). It is therefore crucial to obtain a more holistic idea on how *Alternaria* mycotoxins affect intestinal immune responses.

To better characterize the immunotoxic potential of key *Alternaria* mycotoxins, this study aimed to systematically assess and compare the effects of seven selected compounds, namely AOH, AME, ALT, ALTP, ATX-I, AST and TEN, on inflammatory responses in both immune and intestinal cells. NF-κB activity was measured using a reporter gene assay in THP-1 Lucia™ monocytes and macrophages. In parallel, transcript levels of the pro- and anti-inflammatory cytokines IL-6, IL-8, IL-10 and TNF-α were quantified by qRT-PCR under IL-1β-stimulated conditions in the tumorigenic Caco-2 and non-tumorigenic HCEC-1CT intestinal cell lines. The most promising results were verified by analyzing cytokine secretion using ELISA.

## Materials and methods

### Materials

Cell culture media (Dulbecco’s Modified Eagle Medium (DMEM); Roswell Park Memorial Institute 1640 medium (RPMI 1640) and Medium 199), penicillin streptomycin (P/S) solution, fetal bovine serum (FBS), HEPES buffer and sodium pyruvate were purchased from Thermo Fisher Scientific Inc. (Waltham, USA). Zeocin® and Normocin® were obtained from Invivogen (San Diego, USA), trypsin from Sigma Aldrich (St. Louis, USA), accutase from Merck KGaG (Darmstadt, Germany), insulin-transferase-selenium and gentamicin solutions from Life Technologies Corporation (Carlsbad, USA), human recombinant epidermal growth factor (EGF) and cosmic calf serum® from Corning Inc. (Corning, USA), and hydrocortisone and phorbol 12-myristate 13-acetate (PMA) from Sigma Aldrich (St. Louis, USA). Cell culture consumables were bought from Sarstedt AG + Co. KG (Nuembrecht, Germany), Carl Roth GmbH + Co. KG (Karlsruhe, Germany), VWR International (Pennsylvania, USA) and Henke-Sass, Wolf GmbH (Tuttlingen, Germany). LPS from *Escherichia coli* and dexamethasone (Dexa) were acquired from Sigma Aldrich (St. Louis, USA) while the recombinant human IL-1β and the Quanti-Luc™ reagent were bought from Invivogen (San Diego, USA). The CellTiter-Blue® (CTB) reagent was purchased from Promega Corp. (Fitchburg, USA), and dimethyl sulfoxide (DMSO), Triton X-100, β-mercaptoethanol and TAE buffer from Carl Roth GmbH + Co. KG (Karlsruhe, Germany). The RNeasy Mini Kit and the primer for IL-6, IL-8, IL-10 and TNF-α were obtained from Quiagen N.V. (Hilden, Germany), while the primer for GAPDH were designed and acquired from Eurofins Scientific SE (Luxembourg City, Luxembourg). The High-Capacity cDNA Reverse Transcription Kit, the *Power* SYBR™ Green PCR Master Mix, PCR plates and ELISA Kits (Human IL-6, IL8, IL-10 and TNF-α; uncoated) were purchased from Thermo Fisher Scientific Inc. (Waltham, USA). The *Alternaria* mycotoxins ALT, ATX-I and TEN were acquired from Cayman Chemicals Company (Ellsworth, USA), ALTP from Cfm Oskar Tropitzsch (Marktredwitz, Germany), AOH from Szabo-Scandic (Vienna, Austria), and AST from Molport (Riga, Latvia). AME was synthesized by Prof. R. Süßmuth, TU Berlin, Germany, in the framework of the EU-cofounded PARC project.

### Cell culture

THP-1 Lucia™ monocytes (InvivoGen, San Diego, USA) were routinely cultured in RPMI 1640 medium supplemented with 10% heat-inactivated FBS, 25 mM HEPES, 1% P/S (100 U/ml), and 100 µg/ml Normocin®. To maintain reporter gene stability, cells were cultured under selection pressure by adding 100 µg/mL Zeocin® every second passage. Toxicity testing was performed with THP-1 Lucia™ monocytes and M0 macrophages. For differentiation, monocytes were seeded into 96-well plates at a density of 0.1 × 10^6^ cells/well and treated with 10 ng/ml PMA for 72 h. Macrophages were kept in PMA-free medium for further 24 h. Monocytes were also seeded at a density of 0.1 × 10^6^ cells/well in 96-well plates and used immediately. The tumorigenic epithelial Caco-2 brush border-expressing clone (C2BBe1, ATCC® CRL-2102™) was obtained from the American Type Culture Collection (ATCC, Manassas, USA). Cells were cultured in DMEM containing 4.5 g/L glucose, supplemented with 1 mM sodium pyruvate, 10% heat-inactivated FBS, 0.01 mg/ml insulin-transferrin-selenium and 1% P/S (100 U/ml). For cell culture-based experiments Caco-2 cells were seeded at a density of 8.5 × 10^4^ cells/cm^2^ and were cultivated for 7 days to obtain a confluent and partially differentiated cell monolayer. In the process of differentiation, the growth medium was exchanged every 2–3 days. The non-tumorigenic human colonic epithelial cell line HCEC-1CT was kindly provided by Prof. Jerry W. Shay (UT Southwestern Medical Center, Dallas, USA). Cells were cultured in DMEM containing 4.5 g/L glucose, supplemented with 2% cosmic calf serum® serum, 2% 10 × Medium 199, 20 mM HEPES buffer, 50 µg/mL gentamicin, 1 µg/mL hydrocortisone, 0.01 mg/ml insulin-transferrin-selenium, and 20 ng/mL EGF. Before conducting experiments HCEC-1CT cells were seeded at a density of 1.7 × 10^4^ cells/cm^2^ and cultivated for 48 h to obtain a tight cell monolayer. All cell lines were maintained in a humidified incubator at 37 °C with 5% CO_2_ and passaged twice per week upon reaching approximately 80% confluency. Routine screening for mycoplasma contamination was performed at regular intervals.

### CellTiter-Blue® (CTB) assay

The CTB assay was performed in the applied cell models to evaluate potential cytotoxic effects and to determine non-cytotoxic concentrations of the selected *Alternaria* mycotoxins for subsequent qRT-PCR analyses in Caco-2 and HCEC-1CT cells. The full range of concentrations tested for each mycotoxin in Caco-2 and HCEC-1CT cells in the CTB assay is reported in Table 1, which also shows the concentrations applied in qRT-PCR experiments. For THP-1 cells, monocytes were either used directly or differentiated into macrophages as described in Sect. “Cell culture”. THP-1 monocytes and macrophages were exposed for 2 h to the individual *Alternaria* mycotoxins (AOH, AME, ALT, ATX-I, ALTP, AST and TEN; 0.01–25 µM), solvent control (0.25% DMSO), or negative control (1 µM Dexa), with a final DMSO concentration of 0.25%. Subsequently, to activate the NF-κB pathway, 10 ng/ml LPS were added to all wells except the solvent control, followed by incubation of cells for additional 18 h. Cells exposed to 0.25% DMSO for 2 h and treated with 10 ng/mL LPS served as the positive control. At the end of the incubation period (20 h), the CTB reagent was added to each well (1:10 dilution in supernatant), followed by 2 h incubation. As positive control for cytotoxicity, cells which were previously just incubated with medium, were exposed to 0.01% Triton X-100 for 2 h. Cells were then centrifuged at 140 rcf for 2 min, and 100 µL of the supernatants were transferred to a black 96-well plate for analysis. Caco-2 and HCEC-1CT cells were seeded and cultivated in 96-well plates as described in Sect. “Cell culture”. After a growth period of 7 days (Caco-2) or 48 h (HCEC-1CT), the medium was replaced with fresh medium containing the individual *Alternaria* mycotoxins at various concentrations (ranging from 0.1–200 µM), with a final DMSO concentration of 1%. Following 2 h of exposure, cells were additionally stimulated with 25 ng/ml IL-1β for another 18 h. IL-1β was used to induce an inflammatory response, as these epithelial cell lines are largely unresponsive to LPS, as previously described by Van de Walle et al. (2010). Cells exposed to only 1% DMSO or additionally to 25 ng/mL IL-1β served as the solvent and positive controls, respectively. IL-1β-stimulated cells treated with 1 µM Dexa were used as the negative control. After a total incubation time of 20 h the test media were removed, and cells were washed once with PBS. Cells were incubated for 2 h with a 10% CTB reagent solution (1:10 dilution in DMEM without phenol red). A 0.1% Triton X-100 solution was applied as positive control for cytotoxicity. At the end of the 2 h incubation period of cells with the CTB solution, 80 µl of the supernatants were transferred to a black 96-well plate for analysis. For all cell lines, fluorescence was measured at 560/590 nm (λ_ex_/λ_em_) with a microplate reader (Cytation 3 imaging plate reader) equipped with the software Gen5 (version 3.08; BioTek Instruments, Winooski, USA).

**Table 1:** Overview of the *Alternaria* mycotoxins and the respective concentrations (µM) applied in CTB and qRT-PCR experiments using Caco-2 and HCEC-1CT cells

	Concentrations tested in Caco-2 cells [µM]		Concentrations tested in HCEC-1CT cells [µM]	
	CTB	qRT-PCR	CTB	qRT-PCR
AOH	0.1, 1, 10, 25, 50, 100	0.1, 1, 10, 30	0.1, 1, 10, 25, 50, 100	0.1, 1, 10, 30
AME	0.1, 1, 10, 25, 50, 100	0.1, 1, 10	0.1, 1, 10, 25, 50, 100	0.1, 1, 10
ALT	0.1, 1, 10, 25, 50, 100	0.1, 0.5, 1, 5, 10	0.1, 1, 10, 25, 50, 100	0.1, 1, 5, 10
ALTP	0.1, 1, 5, 10, 25, 50	0.1, 1, 2.5, 5, 10	0.1, 1, 2.5, 5, 10, 25, 50	0.1, 1, 2.5
ATX-I	1, 10, 25, 50, 100, 200	0.1, 1, 10, 20	0.1, 1, 10, 25, 50, 100, 200	0.1, 1, 10, 20
AST	0.1, 1, 10, 25, 50, 100	0.1, 1, 10	0.1, 1, 10, 25, 50, 100	0.1, 1, 10
TEN	0.1, 1, 10, 25, 50, 100	0,01, 0.1, 1, 10	0.1, 1, 10, 25, 50, 100	0.1, 1, 10

### NF-κB reporter gene assay

To evaluate and compare the impact of the individual *Alternaria* mycotoxin on the NF-κB signaling pathway, the NF-κB reporter gene assay was conducted in THP-1 Lucia™ NF-κB monocytes and PMA-differentiated M0 macrophages. The experimental conditions were identical to those described for the CTB assay (see Sect. “CellTiter-Blue® assay”). After a total incubation time of 20 h, NF-κB activity was assessed according to the manufacturer’s instructions by measuring luciferase activity using the Quanti-Luc™ reagent, a coelenterazine-based luminescence substrate. Luminescence was detected with a microplate reader.

### Quantitative real time PCR (qRT-PCR)

To determine and compare the effects of non-toxic concentrations of the seven *Alternaria* mycotoxins of interest on mRNA transcript levels of IL-6, IL-8, IL-10 and TNF-α, two-step qRT-PCR was performed in Caco-2 and HCEC-1CT cell. The complete range of concentrations tested for each compound is shown in Table 1. In particular, cells were seeded and grown in 24 and 12 well plates, respectively, as described in Sect. “Cell culture”. The experimental setup was identical to that described for the CTB assay (see Sect. “CellTiter-Blue® assay”), except that the incubation time was reduced to 5 h and the final DMSO concentration was adjusted to 0.1% for all conditions. This lower concentration was chosen because DMSO has previously been reported to exhibit anti-inflammatory effects in intestinal cells even at low levels (Hollebeeck et al. 2011). At the end of the incubation time the supernatants were collected and stored at − 80 °C for further analysis (see Sect. “Enzyme-linked immunosorbent assay”). Cells were washed with ice-cold PBS, and cell lysis and total RNA extraction were performed using the RNeasy Mini Kit, following the manufacturer’s instructions. The concentration and purity of the extracted RNA was determined with the spectrophotometer NanoDrop 2000 (Thermo Fisher Scientific, Waltham, USA). For each sample, 1 µg total RNA was reverse transcribed into complementary DNA (cDNA) using the High-Capacity cDNA Reverse Transcription Kit in accordance with the manufacturer’s protocol. Gene-specific cDNA was exponentially amplified by qRT-PCR with the QuantStudio 3 PCR System (Thermo Fisher Scientific, Waltham, USA). Reactions were carried out in 96-well plates using *Power* SYBR™ Green PCR Master Mix, along with gene-specific primer assays with a final reaction volume of 20 µL per well. The following primer assays were used for amplification: GAPDH (reverse: GATTTGGTCGTATTGGGCGC, forward: TTCCCGTTCTCAGCCTTGAC), IL-6 (Hs_IL6_1_SG; QT00083720), IL-8 (Hs_CXCL8_1_SG; QT00000322), IL-10 (Hs_IL10_1_SG; QT00041685), TNF-α (Hs_TNF_1_SG; QT00029162). The amplification protocol began with an initial activation step at 95 °C for 15 min, followed by 40 cycles of denaturation at 94 °C for 15 s, annealing at 55 °C for 30 s, and extension at 70 °C for 30 s. A final melting curve analysis was performed to confirm amplification specificity (1 min at 65 °C; heating in 0.5 °C steps to 94 °C, holding at 95 °C for 15 s). Relative mRNA expression levels were calculated using the 2^−ΔΔCT^ method. CT values of target genes were normalized to the average CT values of the housekeeping gene GAPDH. Fold changes were determined by comparison to the corresponding samples of the positive control.

### Enzyme-linked immunosorbent assay (ELISA)

To determine whether the *Alternaria* mycotoxins of interest also impact cytokine secretion, ELISAs were performed. Protein levels of IL-6, IL-8, IL-10, and TNF-α were quantified using commercial kits according to the manufacturer’s instructions. In particular, ELISAs were performed with supernatants collected after cell treatment (see Sect. “Quantitative real time PCR”). However, analyses were restricted to samples from conditions that had previously induced significant changes in mRNA transcript levels.

### Statistical analysis

Statistical analysis was performed with the Origin Pro® 2022 software (OriginLab, Northampton, USA). All cell culture-based experiments were performed in technical triplicates (CTB and NF-κB reporter gene assay) or technical duplicates (qRT-PCR and ELISA) and in at least three biological replicates. Results were normalized to the solvent and positive control (NF-κB reporter gene assay) or only the respective positive control in case of the other assays and are expressed as mean + standard deviation (SD) of the biological replicates. To evaluate significant differences between the controls used for normalization and the test conditions, and between results obtained for monocytes and macrophages or Caco-2 and HCEC-1CT cells Student’s *t*-tests were performed. One-way ANOVA with Fisher-LSD post-hoc test was applied to calculate differences between the various concentrations of one mycotoxin.

## Results

### Immunosuppressive and cytotoxic effects on THP-1 monocytes and M0 macrophages

**Fig. 2:**
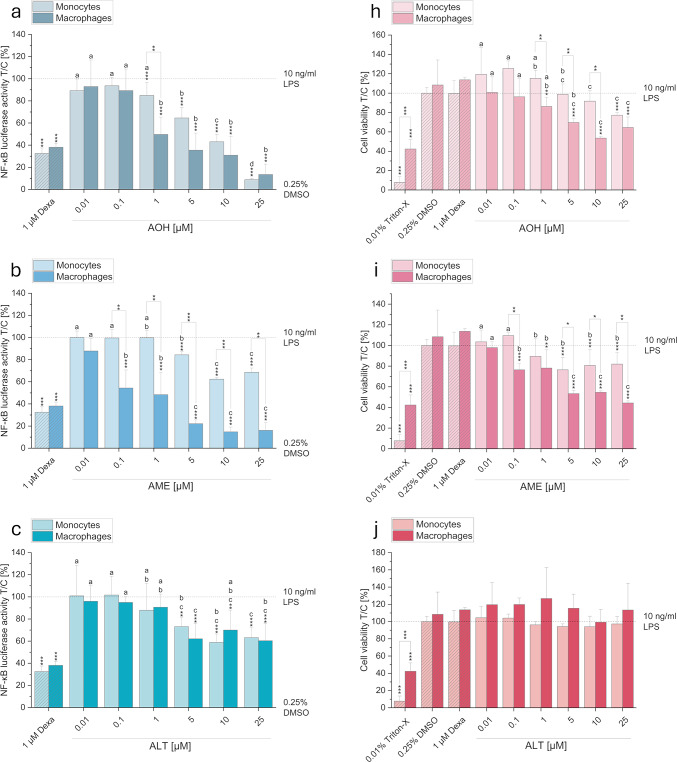

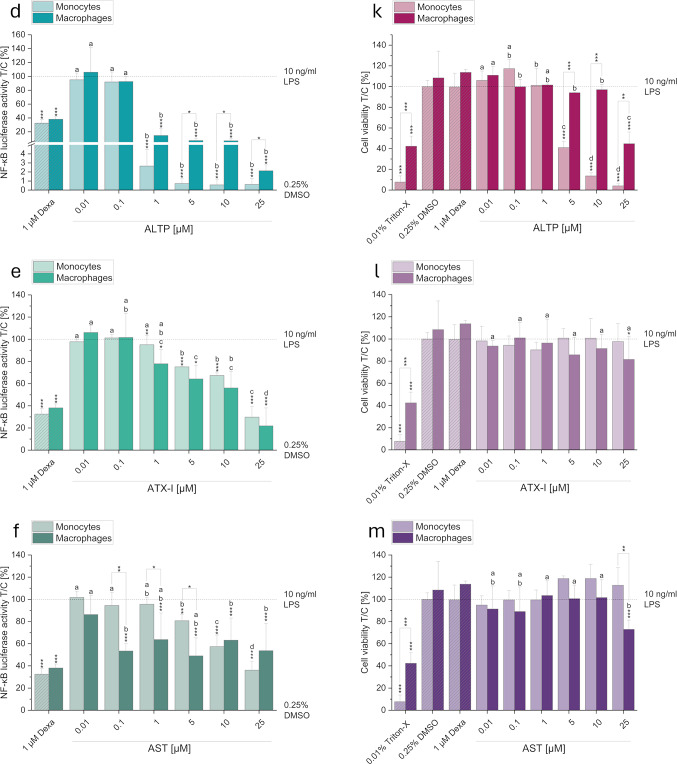

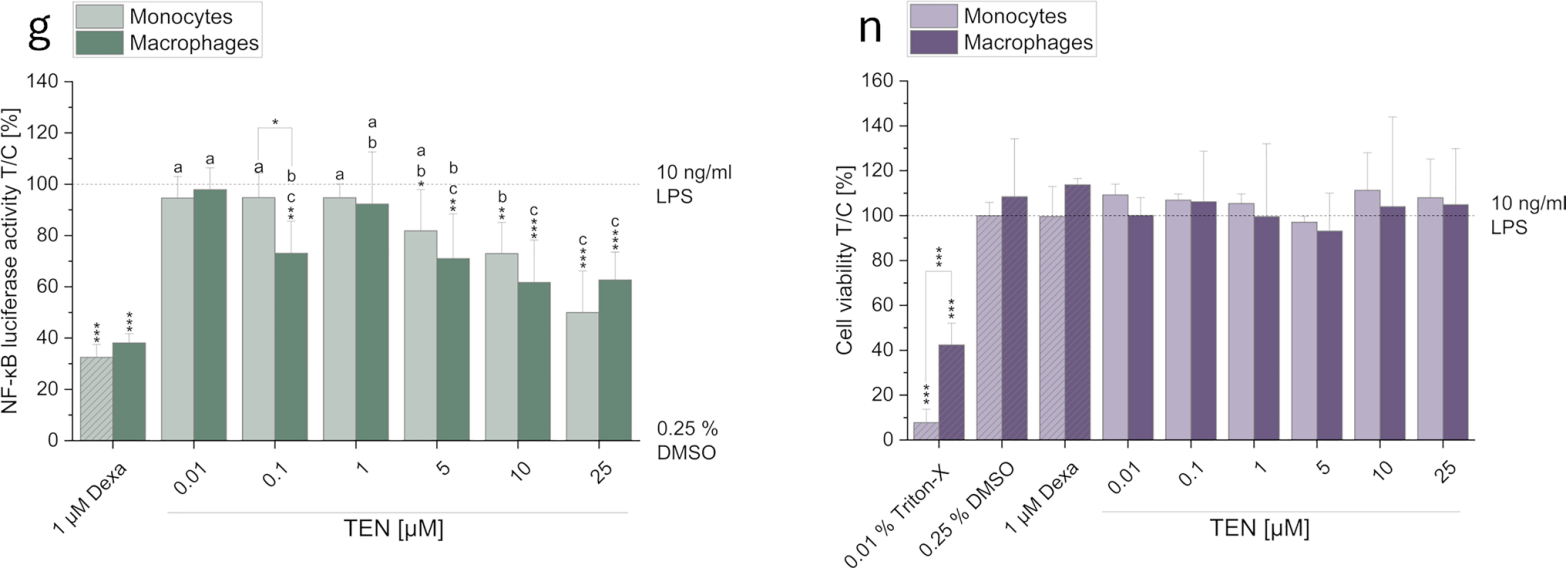
Immunoinhibitory and cytotoxic effects of the *Alternaria* mycotoxins alternariol (AOH), alternariol monomethyl ether (AME), altenuene (ALT), alterperylenol (ALTP), altertoxin I (ATX-I), altersetin (AST), and tentoxin (TEN) in THP-1 Lucia™ monocytes and PMA-differentiated M0 macrophages. Panels **a**–**g** show the results of the NF-κB reporter gene assay performed under co-stimulation with 10 ng/mL lipopolysaccharide (LPS), with 1 µM dexamethasone (Dexa) serving as a negative control. Panels **h-n** depict the effects of mycotoxins on cell viability, as measured by the CellTiter-Blue® (CTB) assay. Triton X-100 (0.01%) was used as a positive control for cytotoxicity. Data are presented as mean + SD of at least three independent experiments. In **a**–**g** results are expressed relative to the solvent control (0.25% DMSO) and positive control (10 ng/mL LPS), while in **h**–**n** results are presented relative only to the positive control, as indicated by dotted lines. Significant differences between the various treatments and the positive control, as well as between monocytes and macrophages under the same treatment condition, were evaluated by applying the Student’s *t*-test (*p < 0.05, **p < 0.01, and ***p < 0.001). One-way ANOVA followed by Fisher-LSD post hoc test (**a**–**d**; *p* < 0.05) was applied to determine statistically significant differences between concentrations of the same mycotoxin

The immunosuppressive potential of the seven selected *Alternaria* mycotoxins (i.e. AOH, AME, ALT, ALTP, ATX-I, AST and TEN) was investigated in THP-1 monocytes and PMA-differentiated M0 macrophages using the NF-κB reporter gene assay. In detail, cells were exposed to six concentrations of each mycotoxin (0.01–25 µM) in the presence of 10 ng/ml LPS to assess their impact on the NF-κB signaling pathway. As shown in Fig. 2a–g all tested mycotoxins significantly suppressed LPS-induced NF-κB activation in both cell types, although with varying potencies. In monocytes AOH, ALTP and ATX-I induced immunosuppressive effects at concentrations ≥ 1 µM (AOH, ALTP—*p* < 0.001; ATX-I—*p* < 0.1), while for AME, ALT, AST, TEN suppressive effects were observed starting at 5 µM (AME—*p* < 0.001; ALT, AST—*p* < 0.01; TEN—*p* < 0.05). Moreover, in macrophages AOH, ALTP and ATX-I also suppressed NF-κB activity at ≥ 1 µM (AOH, ALTP—*p* < 0.001; ATX-I—*p* < 0.05), while ALT was active at concentrations ≥ 5 µM (*p* < 0.001). In contrast to what was observed in monocytes, AME, AST and TEN already exhibited immunosuppressive effects at 0.1 µM (AME, AST—*p* < 0.001; TEN—*p* < 0.01). It should be noted that TEN did not show activity at 1 µM, however, a clear concentration-dependent immunosuppressive effect was measured starting at 5 µM. When comparing the two cell models, statistically significant differences in sensitivity were observed for the respective lowest effect concentrations of AOH, AME, AST and TEN. Furthermore, across the concentration range of 0.1–25 µM, AME exhibited stronger immunosuppressive effects in macrophages and in the range between 0.1 and 5 µM AST was found to be more effective in inhibiting NF-κB activation in macrophages compared to monocytes. In contrast, ALTP induced a more pronounced suppression of NF-κB activity in monocytes at concentrations between 5 and 25 µM.

To exclude experimental artefacts deriving from cytotoxicity, the CTB assay was carried out in parallel (Fig. 2h–n). No reduction in cell viability was observed for ALT and TEN in neither THP-1 monocytes nor PMA-differentiated macrophages. Moreover, AST and ATX-I only exerted mild cytotoxicity in macrophages at 25 µM (AST—*p* < 0.001; ATX-I—*p* < 0.01). On the contrary, exposure of monocytes to AOH lead to minor losses in viable cell at 25 µM, while AME and ALTP showed significant cytotoxicity at concentrations ≥ 5 µM (p < 0.001). At the highest concentration tested, AME reduced viability to approximately 80%, indicating only mild toxicity. In contrast, ALTP led to an almost complete loss of viable monocytes. Of note, the pattern was reversed in M0 macrophages, where ALTP reduced viability of cells to around 45% (*p* < 0.001) only at the highest concentration tested, namely 25 µM. AOH exhibited cytotoxic effects in macrophages starting at 1 µM (*p* < 0.01), whereas AME affected viability already at concentrations as low as 0.1 µM (*p* < 0.001). Statistical comparisons between the two cell types revealed significant differences in cytotoxicity for AME at 0.1 µM and between 5 and 25 µM, as well as for AOH in the rage of 1–10 µM and ALTP between 5 and 25 µM.

### Impact of *Alternaria* mycotoxins on IL-6, IL-8, IL-10 and TNF-α gene transcription in Caco-2 and HCEC-1CT cells

To investigate the ability of *Alternaria* mycotoxins to suppress IL-1β-induced cytokine gene transcription in HCEC-1CT and differentiated Caco-2 cells, changes in mRNA levels of IL-6, IL-8, IL-10, and TNF-α were assessed by qRT-PCR. To ensure that only non-cytotoxic concentrations were tested, CTB assays were performed prior to the gene transcription analysis (see Table 1 for the full range of concentrations tested). As shown in Supplementary Fig. 1, none of the mycotoxins exerted cytotoxic effects at the concentrations applied in qRT-PCR experiments.

**Fig. 3:**
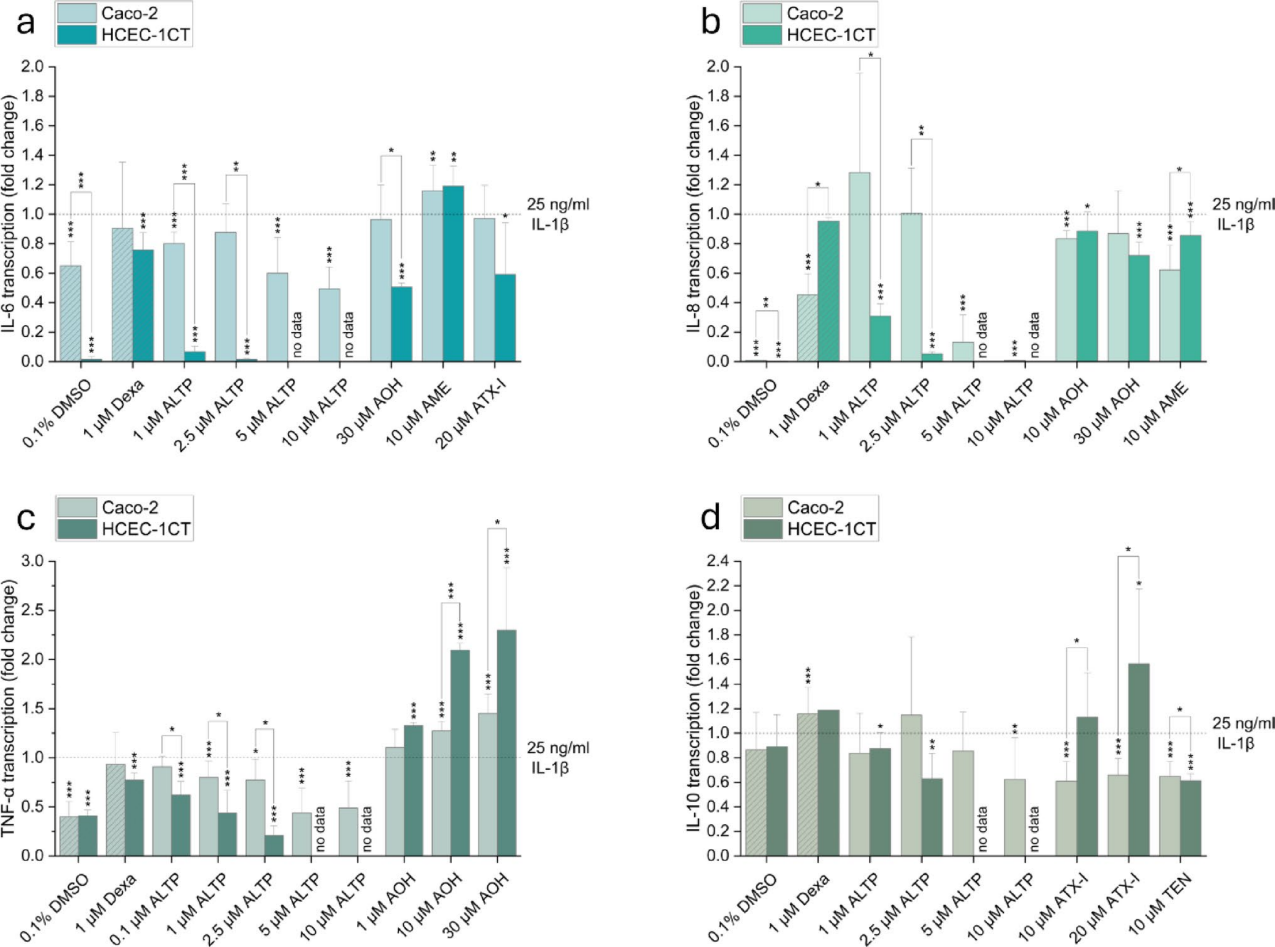
Impact of the *Alternaria* mycotoxins alterperylenol (ALTP), alternariol (AOH), alternariol monomethyl ether (AME), altertoxin I (ATX-I) and tentoxin (TEN) on the relative gene transcription of IL-6, IL-8, IL-10 and TNF-α in IL-1ß stimulated Caco-2 and HCEC-1CT cells. Cells were pre-incubated with non-toxic concentrations of the respective mycotoxins or 1 µM dexamethasone (Dexa; used as a negative control) for 2 h, followed by stimulation with 25 ng/mL IL-1β for an additional 3 h. Gene expression changes were examined by qRT-PCR and results were calculated as relative gene transcription (2^−ΔΔCT^), normalized to GAPDH and compared to the positive control (25 ng/ml IL-1ß), as indicated by dotted lines. Data are presented as mean + SD from at least three independent experiments. Statistical significances between mycotoxin-treated samples and the positive control, and between Caco-2 and HCEC-1CT cells under the same treatment condition were evaluated using the Student’s *t*-test (*p < 0.05, **p < 0.01, and ***p < 0.001)

The assessment of cytokine gene expression levels, performed after 5 h of incubation, revealed the ability of the various mycotoxins under investigation to modulate cytokine expression in distinct ways. The complete dataset covering both cell lines, all seven mycotoxins, and the four cytokines is provided in the supplementary materials (Supplementary Figs. 2 and 3). As shown in Fig. 3a–d, which reports the most relevant results obtained, the mycotoxin ALTP significantly downregulated transcript levels of all four cytokines in both cell lines. In detail, in HCEC-1CT cells transcription of IL-6, IL-8 and IL-10 was reduced starting at a concentration of 1 µM (IL-6, IL-8—*p* < 0.001; IL10—*p* < 0.05), whereas TNF-α expression was already affected at 0.1 µM (*p* < 0.001). Notably, exposure to 2.5 µM ALTP led to an almost complete suppression of IL-6 and IL-8 expression. Due to cytotoxic effects, higher concentrations could not be tested (Supplementary Fig. 3). In comparison, exposure of Caco-2 cells to ALTP resulted in reduced mRNA levels of IL-6 and TNF-α at 1 µM (*p* < 0.001), of IL-6 at 5 µM (*p* < 0.001) and of IL-10 at 10 µM (*p* < 0.01). The most striking effect was observed for IL-8, where 10 µM ALTP led to an almost complete suppression of gene expression, reducing it at a level comparable to the solvent control (0.1% DMSO). With respect to the other mycotoxins, exposure of HCEC-1CT cells to 30 µM AOH (*p* < 0.001) and 20 µM ATX-I (*p* < 0.05) lead to a reduction in IL-6 transcript levels, while no effects were observed in Caco-2 cells (Fig. 3a). Interestingly, treatment with 10 µM AME induced a minor but statistically significant upregulation of IL-6 (*p* < 0.01) in Caco-2 and HCEC-1CT cells (Fig. 3a). On the contrary, the same concentration of AME (10 µM) significantly decreased IL-8 mRNA levels in both intestinal cell lines (*p* < 0.001), with a more pronounced effect measured in Caco-2 cells (Fig. 3b). Of note, slight but significant downregulations of IL-8 expression were also observed upon exposure of both HCEC-1CT (*p* < 0.05) and Caco-2 cells (*p* < 0.001) to 10 µM AOH, as well as following treatment of HCEC-1CT cells with 30 µM AOH (Fig. 3b). A distinct pattern emerged for the gene transcription of TNF-α. In HCEC-1CT cells, exposure to AOH at concentrations ranging from 1 to 30 µM resulted in a significant upregulation of TNF-α mRNA levels (*p* < 0.001), whereas in Caco-2 cells, this effect was restricted to 10 and 30 µM (*p* < 0.001, Fig. 3c). Regarding the transcript levels of IL-10, treatment with 10 µM TEN significantly decreased transcript levels in both cell lines (*p* < 0.001). Finally, in Caco-2 cells ATX-I caused a downregulation of IL-10 gene transcription at 10 and 20 µM (*p* < 0.001), whereas exposure of HCEC-1CT cells to 20 µM ATX-I led to a moderate but statistically significant upregulation (*p* < 0.05; Fig. 3d).

### Impact of *Alternaria* mycotoxins on IL-6, IL-8 and TNF-α protein levels in Caco-2 and HCEC-1CT cells

**Fig. 4:**
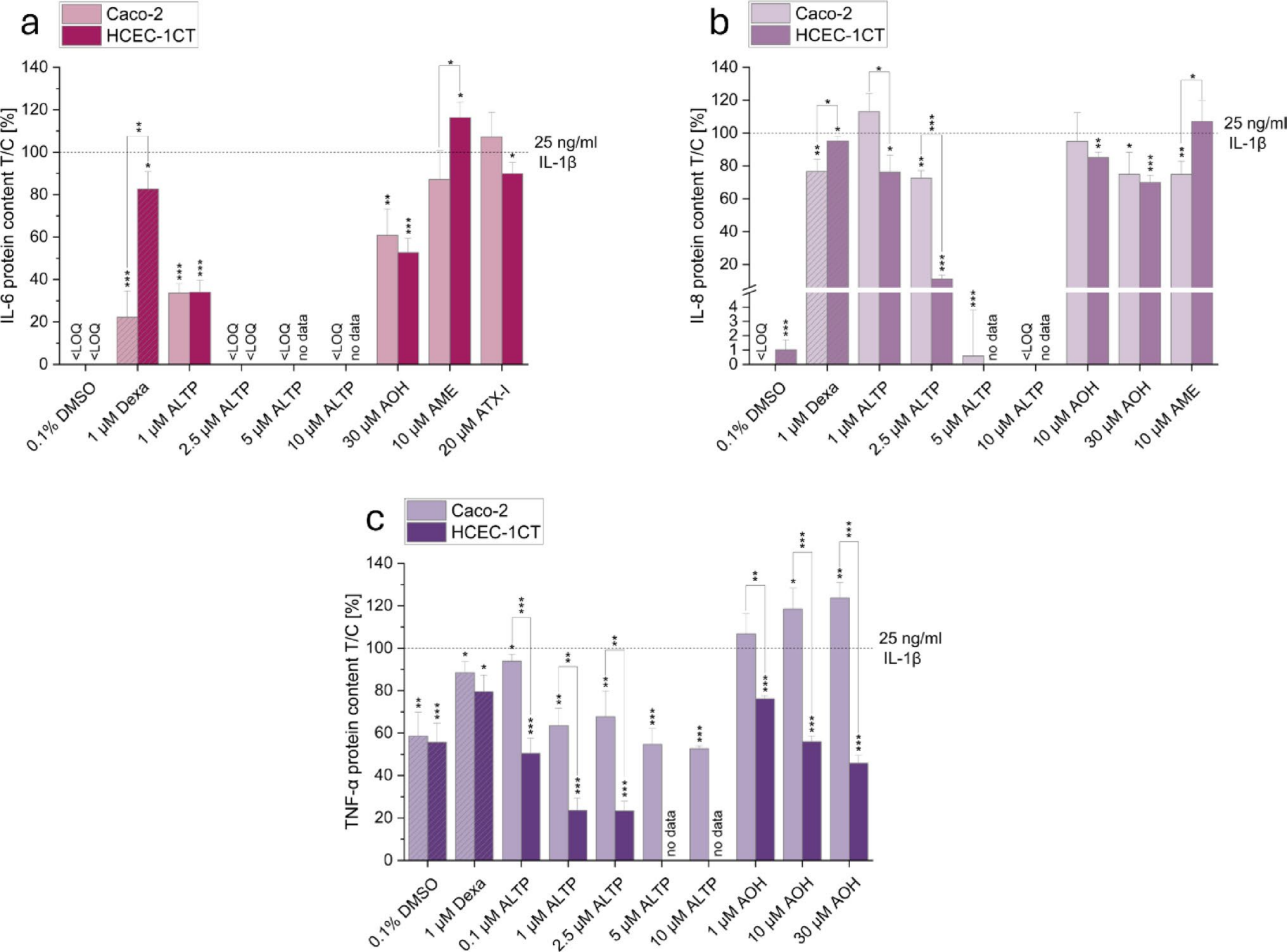
Impact of the *Alternaria* mycotoxins alterperylenol (ALTP), alternariol (AOH), alternariol monomethyl ether (AME), altertoxin I (ATX-I) and tentoxin (TEN) on protein levels of IL-6, IL-8 and TNF-α in IL-1ß stimulated Caco-2 and HCEC1CT cells. Cells were pre-incubated with non-toxic concentrations of mycotoxins or 1 µM dexamethasone (Dexa; used as negative control) for 2 h, followed by stimulation with 25 ng/mL IL-1β for an additional 3 h. Cytokine secretion was measured in cell culture supernatants using ELISA, and results were normalized to the positive control (25 ng/ml IL-1β), as indicated by dotted lines. Data are presented as mean + SD from at least three independent experiments. Statistical significances between mycotoxin-treated samples and the positive control, and between Caco-2 and HCEC-1CT cells under the same treatment condition were evaluated using the Student’s *t*-test (*p < 0.05, **p < 0.01, and ***p < 0.001)

Based on the qRT-PCR results, selected treatment conditions that caused significant changes in cytokine gene transcription, were further analyzed for their potential to alter cytokine secretion. To this end, cell culture supernatants collected after 5 h of incubation were analyzed by ELISAs to quantify protein levels of IL-6, IL-8, IL-10, and TNF-α. Because IL-10 levels were below the detection limit in all tested conditions, quantification by this method was not possible (data not shown). As shown in Fig. 4a–c, in Caco-2 and HCEC-1CT cells, ALTP significantly reduced the secretion levels of the three cytokines IL-6, IL-8 and TNF-α. In both cell lines IL-6 secretion was significantly reduced starting at 1 µM (*p* < 0.001), and levels fell below the limit of quantification (LOQ) at 2.5 µM (Fig. 4a). In comparison, IL-8 levels were decreased at concentrations ≥ 2.5 µM (*p* < 0.01) and ≥ 1 µM (*p* < 0.05) in Caco-2 and HCEC-1CT cells, respectively, with the IL-8 level falling below the LOQ at 10 µM. Furthermore, in both intestinal cell lines, exposure to ALTP at a concentration as low as 0.1 µM already resulted in a significant reduction of TNF-α content (Caco-2—*p* < 0.05; HCEC-1CT—*p* < 0.001), with a stronger effect detected in HCEC-1CT cells (Fig. 4c). IL-6 levels were reduced in both cell lines following exposure to 30 µM AOH (Caco-2—*p* < 0.01; HCEC-1CT—*p* < 0.001), and in HCEC-1CT cells to 20 µM ATX-I (*p* < 0.05). Surprisingly, while exposure of HCEC-1CT cells to 10 µM AME resulted in a significant increase in IL-6 secretion (*p* < 0.05), no effects were observed in Caco-2 cells (Fig. 4a). AOH and AME also affected IL-8 levels. In detail, 10 µM and 30 µM AOH lead to a reduction of the cytokine levels in HCEC-1CT cells (*p* < 0.01) and Caco-2 cells (*p* < 0.001), respectively. Additionally, exposure to 10 µM AME decreased the levels of IL-8 only in Caco-2 cells (*p* < 0.01; Fig. 4b). Interestingly, while an increased TNF-α secretion was observed in Caco-2 cells exposed to AOH concentrations ≥ 10 µM, opposite effects were induced by the mycotoxin in HCEC-1CT cells, where significant decreases occurred within the 1–30 µM concentration range (*p* < 0.001, Fig. 4c).

## Discussion

Mycotoxins produced by the *Alternaria* genus represent an emerging concern in food safety due to their frequent occurrence in food and feed and their wide spectrum of adverse effects (Louro et al. 2024). While genotoxic and cytotoxic effects of individual *Alternaria* toxins have been extensively studied, there is still a lack of data regarding their immunomodulatory properties. Thus, this study aimed to address this data gap by systematically comparing the immunosuppressive effects of seven structurally diverse *Alternaria* mycotoxins-AOH, AME, ALT, ALTP, ATX-I, AST, and TEN-on both immune and intestinal epithelial cells.

The results obtained clearly demonstrate the ability of most of the Alternaria mycotoxins tested to significantly inhibit LPS-induced NF-κB activation in THP-1 monocytes and macrophages, albeit with differing potencies (Fig. 2a–g). The only exception was AME, which suppressed NF-κB activity in monocytes and macrophages (Fig. 2b) only at concentrations that also impaired cell viability (Fig. 2i), and thus further investigation to determine its relevance as an immunosuppressive compound are required. In contrast, AOH, ALTP, and ATX-I exerted immunosuppressive effects at concentrations as low as 1 µM in both monocytes and macrophages (Fig. 2a, d and e), and importantly, at non-cytotoxic concentrations (Fig. 2h, k and l). Of note, AST and TEN exhibited higher immunosuppressive potency in macrophages compared to monocytes, with significant effects already observed at concentrations as low as 0.1 µM (Fig. 2f and g). Additionally, ALT showed comparable inhibitory effects in both cell types, with suppression of LPS-induced NF-κB activation starting at 5 µM (Fig. 2c). Some of these findings are in line with previous reports. For instance, Kollarova et al. (2018) and Crudo et al. (2025) demonstrated the immunosuppressive effects of AOH in both monocytes and macrophages at concentrations ≥ 1 µM. Similarly, although data are only available in monocytes, the ability of ALTP, ATX-I, and AST to modulate the NF-κB pathway has also been reported (Crudo et al. 2025; Partsch et al. 2025; Kollarova et al. 2018). The concentrations at which immunosuppressive effects became evident in the present study are consistent with the previous findings of Crudo et al. (2025) and Partsch et al. (2025) (i.e. ≥ 1 µM for ATX-I and ALTP and ≥ 2 µM for AST). Apart from these observations, no additional information on the immunosuppressive properties of other *Alternaria* mycotoxins, nor any direct comparison between monocytes and macrophages, which would help to better characterize the hazard posed by this class of compounds, is currently available. Importantly, the present work provides clear evidence that, in addition to the previously investigated toxins, the *Alternaria* mycotoxins ALT and TEN also target the NF-κB pathway in both monocytes and macrophages. Even though TEN already exerted minor immunoinhibitory activity at 0.1 µM in macrophages, no significant effect was detected at 1 µM, which may be due to the relatively high standard deviation at this concentration. Nevertheless, both compounds showed clear and consistent activity at concentrations ≥ 5 µM in both cell types (Fig. 2c and g), suggesting their identification as immunosuppressive *Alternaria* mycotoxins and highlighting their potential relevance in modulating innate immune responses. Overall, these findings indicate that the impact of immunosuppression is not only compound-specific, but also strongly influenced by the differentiation status of the immune cells.

Of note, as shown in Fig. 2a–g, a general tendency of the *Alternaria* mycotoxins tested to exert stronger immunosuppressive effects in macrophages was observed, with some exceptions. Although dedicated studies are required to elucidate the underlying molecular mechanisms, these stronger effects may be explained by the phenotypic changes associated with the differentiation process. During infection, monocytes migrate into extravascular compartments and differentiate into tissue macrophages, a process accompanied by enhanced pro-inflammatory gene expression, increased receptor abundance, and elevated basal NF-κB signaling (Forrester et al. 2018). In particular, macrophages display higher levels of CD14, which functions as a TLR4 co-receptor and enhances the efficiency and sensitivity of TLR4 activation (Wright et al. 1990; Forrester et al. 2018). They also express higher levels of myeloid differentiation primary response 88 (MyD88), a key adaptor molecule that bridges TLRs to downstream kinases, ultimately activating the NF-κB pathway (Kim et al. 2022). Among others, these up-regulation events underly the stronger responsiveness of macrophages to LPS (Kim et al. 2022). In addition, differentiation is associated with increased cytoplasmic NF-κB accumulation, resulting in faster and more pronounced nuclear translocation following LPS stimulation (Takashiba et al. 1999). Thus, it cannot be excluded that the stronger immunosuppressive effects observed for AOH, AME, AST, and TEN in differentiated THP-1 macrophages reflect the higher abundance of molecular targets available for interference, ultimately leading to more pronounced immunosuppression. By contrast, the lower baseline activity in monocytes may limit the extent of inhibition. These findings highlight the importance of considering both macrophages and their monocytic precursors when assessing the immunotoxic potential of xenobiotics, given the possible increased sensitivity of differentiated immune cells.

It should be noted that immunosuppressive effects may arise not only from the inhibition of key immune-related pathways, such as NF-κB, but also from interference with the differentiation process itself. In this context, Solhaug et al. (2016) and Gufler et al. (2025) both reported that co-incubation of monocytes with AOH and PMA -which is used to induce differentiation-leads to altered expression of differentiation markers, such as CD14 and CD11b, and impairs the acquisition of macrophage-like morphology.

While direct inhibition of the NF-κB pathway and interference with differentiation are clear indicators of immunosuppressive potential, cytotoxic effects on immune cells may also contribute to altered responsiveness to inflammatory stimuli. Consistently, the results of the present study demonstrate that the tested mycotoxins can exert cytotoxicity in both cell types, albeit with some differences between monocytes and macrophages. While macrophages displayed signs of cytotoxicity at lower toxin concentrations, monocytes were generally more tolerant. In macrophages cytotoxic effects by AOH and AME were detected at 1 µM and 0.1 µM, respectively, whereas in monocytes the onset of cytotoxicity was observed at 25 µM and 5 µM (Fig. 2h and i). Regarding AST, cytotoxicity was measured only in macrophages exposed to 25 µM (Fig. 2m). The increased vulnerability of macrophages to mycotoxin exposure may be linked to their higher metabolic activity, increased mitochondrial and lysosomal content, and elevated ROS production, which collectively could make them more susceptible to toxin-induced stress and cell death (Daigneault et al. 2010; Ponath and Kaina 2017). Interestingly, an inverse trend was observed for ALTP, where macrophages showed higher tolerance than monocytes, with cytotoxicity occurring at 25 µM and 5 µM, respectively (Fig. 2k). These results are, on the other hand, consistent with previous studies reporting that monocytes are more susceptible to apoptosis and, due to the fact that they are less capable of counteracting oxidative stress (Daigneault et al. 2010). Overall, these findings indicate that the susceptibility of immune cells to *Alternaria* mycotoxins is both compound and cell type dependent and highlight the need for further studies to clarify the mechanisms underlying these differences and to assess their relevance under physiological exposure conditions.

While immune cells are critical mediators of the host’s response to xenobiotics, their activation is among others tightly regulated by signals from IECs. These cells not only represent the primary barrier to ingested toxins but are also involved in the crosstalk with immune cells. Interactions between IECs and immune cells are essential for mucosal barrier integrity, and their dysregulation may contribute to dysbiosis, inflammation, and tumorigenesis (Yao et al. 2024). Considering the central role of the NF-κB pathway in both cell types, assessing the immunomodulatory effects of mycotoxins on IECs is essential to better understand their impact on intestinal immune regulation. Thus, the influence of the selected *Alternaria* mycotoxins on the transcription and protein level of several cytokines in IL-1β stimulated Caco-2 and HCEC-1CT cells was assessed. ALTP caused a downregulation of the transcription and secretion of the three anti-inflammatory cytokines IL-6, IL-8 and TNF-α in both cell lines, suggesting immunosuppressive activity (Fig. 3a–c; Fig. 4a–c). This effect is likely linked to the ability of the perylene quinone to inhibit NF-κB signaling-a mechanism demonstrated in this study for monocytes and M0 macrophages, and also previously described in monocytes by Crudo et al. (2025). Activation of the NF-κB pathway is not limited to the binding of LPS to the pattern recognition receptor TLR4; it can also be triggered by cytokines such as IL-1β, as used in the present study to stimulate IECs. Specifically, the binding of IL-1β to its receptor, the interleukin-1 receptor type 1 (IL-1R1), initiates a MyD88-dependent signaling cascade. This involves activation of the IκB kinase (IKK) complex, which mediates the degradation of the inhibitory protein IκBα. The release of NF-κB from IκBα allows its translocation into the nucleus, where it induces transcription of cytokines harboring NF-κB promoter elements. Suppression of this pathway by ALTP could therefore plausibly explain the observed downregulation of cytokines (Libermann and Baltimore 1990; Hiscott et al. 1993). Nevertheless, the precise molecular mechanism underlying ALTP-mediated inhibition of NF-κB remains to be elucidated.

Apart from ALTP, changes in cytokine levels were also observed upon exposure of the two intestinal cell lines to AOH. In particular, the mycotoxin exerted a milder and more variable effect than ALTP. In HCEC-1CT cells, AOH reduced IL-6 and IL-8 expression at both the transcriptional and protein levels at 30 µM, whereas in Caco-2 cells, these cytokines were less affected (Fig. 3a and b; Fig. 4a and b). In contrast, TNF-α transcription was increased in both cell lines (Fig. 3c), with stronger effects in the non-cancerous HCEC-1CT cells. Interestingly, this upregulation was accompanied by opposing effects at the protein level, as AOH increased TNF-α secretion in Caco-2 but decreased it in HCEC-1CT cells, indicating a cell type-specific post-transcriptional regulation of this cytokine (Fig. 4c). These results are partly in line with Schmutz et al. (2019), who reported a similar TNF-α upregulation alongside reduced IL-6 and IL-8 mRNA levels in Caco-2 cells. While the authors were unable to detect IL-6 and TNF-α at the protein level under exposure conditions similar to those of the present study, their observed reduction in IL-8 secretion aligns with the findings reported here. Consistently, Groestlinger et al. (2022) reported a reduction in IL-8 expression at both the transcriptional and protein levels in HCEC-1CT cells. It should be noted that the authors observed a decrease in TNF-α gene expression, which was not observed in the data presented here. Instead, the present data demonstrate that the mycotoxin induced a comparable reduction in TNF-α secretion, again suggesting post-transcriptional regulatory effects. Furthermore, the reduced IL-6 and IL-8 expression observed in the present study aligns with previous findings, which showed AOH to downregulate LPS-induced IL-6 and IL-8 transcription in THP-1-derived macrophages and BEAS-2B epithelial cells (Grover and Lawrence 2017; Kollarova et al. 2018), further supporting its role as a modulator of inflammatory signaling.

In several studies, changes in the microRNA (miRNA) profile have been suggested as a possible molecular mechanism underlying the immunosuppressive effects of AOH. MiRNAs are small non-coding RNAs that bind to target mRNAs, thereby regulating gene expression post-transcriptionally and ultimately influencing various biological processes, including immune responses and inflammation (O’Neill et al. 2011). Previously, AOH has been shown to potently upregulate miR-16, miR-125b and miR-155 in Caco-2 cells, while simultaneously downregulating miR-146a (Schmutz et al. 2019). In contrast, Kollarova et al. (2018) observed only an upregulation of miR-155 and a downregulation of miR-146a in THP-1 macrophages, whereas miR-16 and miR125b were unaffected by the exposure to AOH. Several miRNAs can modulate TLR and IL-1R signaling by targeting key adaptor proteins, thereby fine-tuning downstream NF-κB-mediated cytokine production. In this context, there might be a direct link between the upregulation of miR-16 and the decrease in IL-6 and IL-8 observed in the present study, as miR-16 is known to target pro-inflammatory cytokines and suppress NF-κB signaling, resulting in downregulation of these cytokines (Li et al. 2021; Zhou et al. 2012). Conversely, miR-146a is generally associated with negative regulation of NF-κB, which can reduce IL-6 and IL-8 secretion, whereas miR-155 typically acts as a positive regulator of NF-κB, promoting pro-inflammatory cytokine expression (Bhaumik et al. 2009; Taganov et al. 2006; Tili et al. 2007). Therefore, the observed downregulation of miR-146a and upregulation of miR-155 by Schmutz et al. (2019) and Kollarova et al. (2018) are contradictory to the reduced IL-6 and IL-8 levels measured in the current study and previously reported by Schmutz et al. (2019) and Groestlinger et al. (2022). However, this could instead contribute to the observed induction of TNF-α, which is also regulated by miRNAs like miR-146a (Nahid et al. 2009). Finally, apart from potential alterations in miRNAs, the changes in cytokine levels observed in this study might also have resulted from direct inhibition of the NF-κB signaling pathway by AOH. This is supported by Groestlinger et al. (2022), who reported reduced nuclear translocation of NF-κB/p65 in IL-1 β stimulated HCEC-1CT cells after the exposure to 10 µM AOH.

Similarly to AOH, the *Alternaria* mycotoxin AME was previously shown to suppress IL-6 and IL-8 secretion in BEAS-2B cells (Grover and Lawrence 2017). However, in the present study, only minor changes were observed, with the mycotoxin slightly increasing IL-6 secretion in HCEC-1CT cells and reducing IL-8 in Caco-2 cells at 10 μM (Fig. 4a and b). As for the other tested mycotoxins, no significant changes in gene transcription of any of the tested cytokines that were reflected at the protein level, as measured by ELISA, were observed, indicating a lack of pronounced immunosuppressive effects in IECs.

As for IL-10, which is generally associated with immunosuppression (Mittal et al. 2015), only small transcriptional decreases were measured for ALTP and TEN in both cell lines, while the effects caused by ATX-I were inconsistent between the two IEC models with an upregulation measured in HCEC-1CT and a downregulation in Caco-2 cells respectively (Fig. 3d). Of note, macrophage studies have reported an AOH-induced increase in IL-10 transcription (Kollarova et al. 2018). Contradictory, in this work AOH did not have an impact on the transcription levels of the immunoinhibitory cytokine. Furthermore, IL-10 content could not be detected in either cell line with the applied ELISA method and therefore results could not be confirmed on a protein level.

Taken together, these results reveal substance-dependent and cell type–specific differences in response to mycotoxin exposure, particularly between immune cells and IECs. In detail, monocytes and macrophages exhibited more pronounced responses to the immunoinhibitory *Alternaria* mycotoxins, reflecting their role in the body’s defense machinery and their priming for rapid inflammatory reactions. In contrast, the reduced susceptibility of IECs is likely attributable to their emphasis on preserving barrier integrity and tightly controlling immune signaling, including the maintenance of low baseline NF-κB activity to avoid unwarranted inflammation in the gut lumen (Wullaert et al. 2011).

## Conclusion

This study provides a comprehensive analysis of the immunosuppressive potential of seven structurally diverse *Alternaria* mycotoxins (i.e. alternariol, alternariol monomethyl ether, altenuene, alterperylenol, altertoxin I, altersetin, and tenuazonic acid) in immune and intestinal epithelial cell models. The findings clearly demonstrate that the investigated mycotoxins exert immunosuppressive effects that are both compound-specific and cell type–dependent, with PMA-differentiated THP-1 macrophages showing higher sensitivity than monocytes, and non-tumorigenic HCEC-1CT cells exhibiting different cytokine response patterns than tumorigenic Caco-2 cells. Overall, these results emphasize the complexity of *Alternaria* mycotoxin–host interactions and underscore the need for further studies to elucidate the molecular mechanisms underlying the cell type–specific immunomodulation. At the same time, the study provides valuable insights for closing existing data gaps and advancing the understanding of how *Alternaria* mycotoxins may modulate both systemic and intestinal immune responses.

## Supplementary Information

Below is the link to the electronic supplementary material.

Supplementary Material 1

## Data Availability

The datasets generated during and/or analysed during the current study are available from the corresponding author on reasonable request.

## Abbreviations

ALT: Altenuene
AOH: Alternariol
AME: Alternariol monomethyl ether
ALTP: Alterperylenol
AST: Altersetin
ATX-I: Altertoxin I
CTB: CellTiter Blue
Dexa: Dexamethasone
ELISA: Enzyme-linked immunosorbent
IL: Interleukin
ICE: Intestinal epithelial cell
LPS: Lipopolysaccharide
miRNA: MicroRNA
NF-κB: Nuclear factor kappa-light-chain-enhancer of activated B cells
PMA: Phorbol 12-myristate 13-acetate
qRT-PCR: Quantitative real time PCR
TEN: Tentoxin
TNF-α: Tumor necrosis factor-alpha
