# Supplementary material for: Immunomodulatory potential of Alternaria mycotoxins on immune and intestinal cells: a comparative systematic in vitro study
Source: Arch Toxicol. 2026 Apr 28;100(8):3519–34. doi: 10.1007/s00204-026-04346-7 (PMC13379405; doi:10.1007/s00204-026-04346-7)
Supplement: Supplementary file 1 — Supplementary Material 1 [file 204_2026_4346_MOESM1_ESM.docx]

**Immunomodulatory potential of *Alternaria* mycotoxins on immune and intestinal cells:
a comparative systematic *in vitro* study**

Vanessa Partsch^1,2^, Amina Selimagić^1^, Francesco Crudo^1^, Doris Marko^1^

^1^University of Vienna, Faculty of Chemistry, Department of Food Chemistry and Toxicology, 1090 Vienna, Austria

^2^University of Vienna, Faculty of Chemistry, Doctoral School in Chemistry, 1090 Vienna, Austria

**Corresponding authors:**

Dr. Francesco Crudo

Department of Food Chemistry and Toxicology, University of Vienna

Währinger Str. 38, 1090 Vienna, Austria

francesco.crudo@univie.ac.at


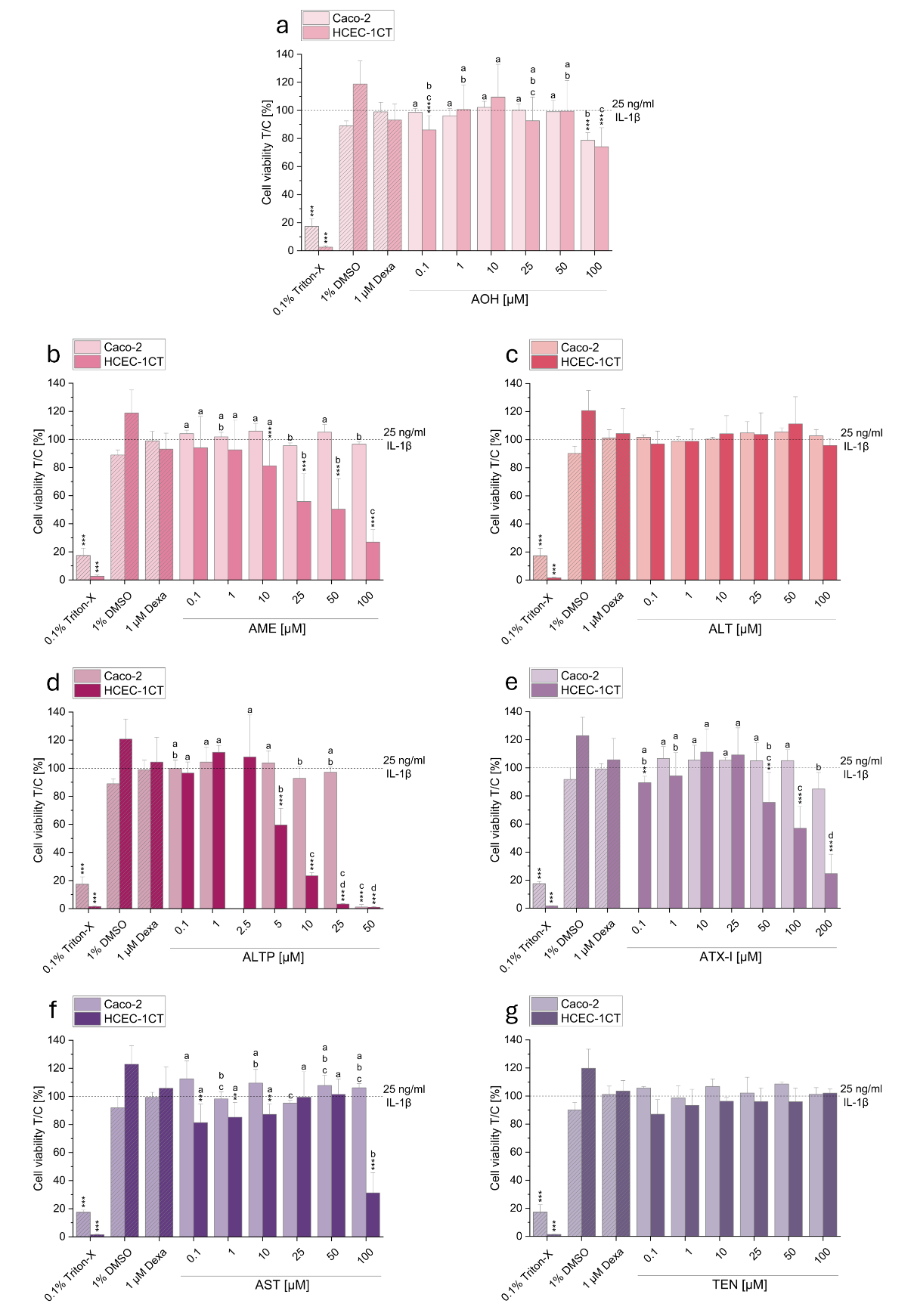


**Supplementary Fig. 1** Cytotoxic effects of the seven *Alternaria* mycotoxins alternariol (AOH; **a**), alternariol monomethyl ether (AME; **b**), altenuene (ALT; **c**), alterperylenol (ALTP; **d**), altertoxin I (ATX-I; **e**), altersetin (AST; **f**), and tentoxin (TEN; **g**) on Caco-2 and HCEC-1CT cells assessed with the CellTiter-Blue® (CTB) assay. Cells were pre-incubated with non-toxic concentrations of the respective mycotoxins or 1 µM dexamethasone (Dexa; used as a control) for 2 h, followed by stimulation with 25 ng/mL IL-1β for an additional 3 h. Triton X-100 (0.01 %) was used as a positive control for cytotoxicity. Data are presented as mean + SD from at least three independent experiments. Results are expressed relative to the positive control (25 ng/mL IL-1β), indicated by dotted lines. Statistical significances between mycotoxin-treated samples and the positive control were evaluated using the Student’s *t*-test (*p<0.05, **p<0.01, and ***p<0.001). One-way ANOVA followed by a Fisher-LSD post hoc test (a-d; p<0.05) was applied to determine statistically significant differences between concentrations of the same mycotoxin


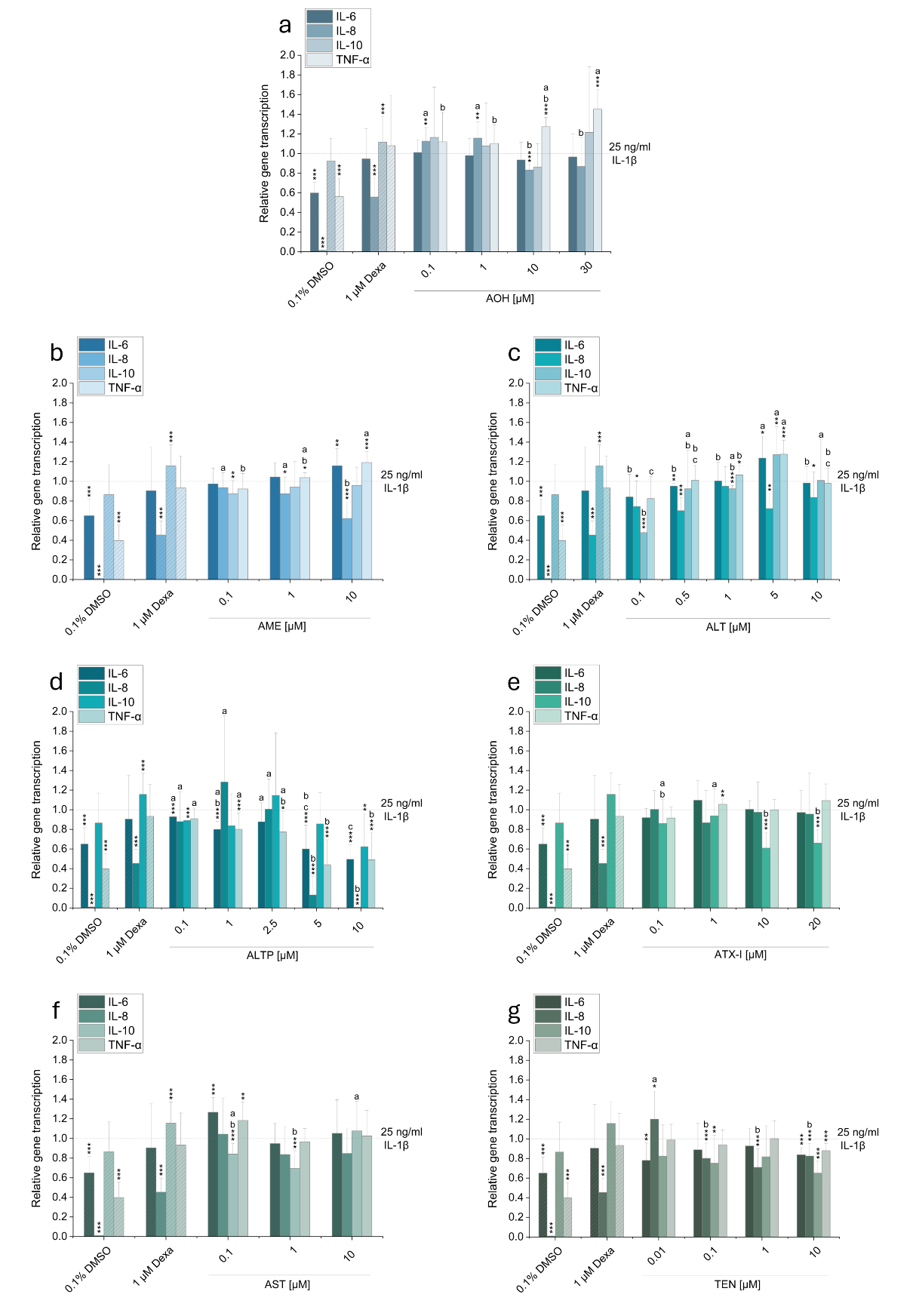


**Supplementary Fig.** **2** Impact of the seven *Alternaria* mycotoxins alternariol (AOH; **a**), alternariol monomethyl ether (AME; **b**), altenuene (ALT; **c**), alterperylenol (ALTP; **d**), altertoxin I (ATX-I; **e**), altersetin (AST; **f**), and tentoxin (TEN; **g**) on relative gene transcription levels of IL-6, IL-8, IL-10 and TNF-α in IL-1ß stimulated Caco-2 cells. Cells were pre-incubated with non-toxic concentrations of the respective mycotoxins or 1 µM dexamethasone (Dexa; used as negative control) for 2 h, followed by stimulation with 25 ng/mL IL-1β for an additional 3 h. Gene expression changes are presented as mean + SD of at least three biological replicates, calculated as relative gene transcription (2^−ΔΔCT^), normalized to GAPDH, and compared to the positive control (25 ng/ml IL-1ß). Statistical significances between mycotoxin-treated samples and the positive control were evaluated using the Student’s *t*-test (*p<0.05, **p<0.01, and ***p<0.001). One-way ANOVA followed by a Fisher-LSD post hoc test (a-d; p<0.05) was applied to determine statistically significant differences between concentrations of the same mycotoxin


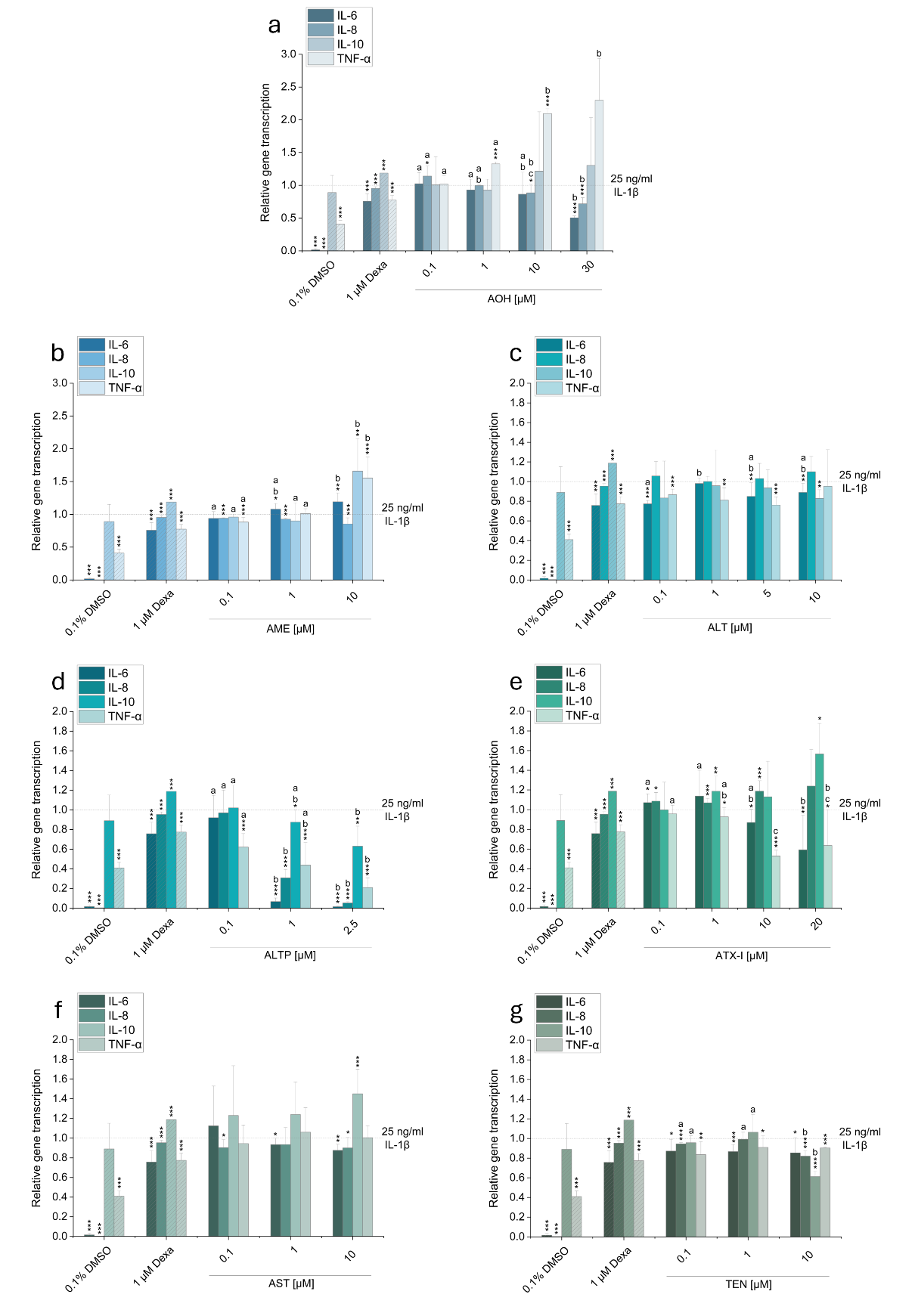


**Supplementary Fig. 3** Impact of the seven *Alternaria* mycotoxins alternariol (AOH; **a**), alternariol monomethyl ether (AME; **b**), altenuene (ALT; **c**), alterperylenol (ALTP; **d**), altertoxin I (ATX-I; **e**), altersetin (AST; **f**), and tentoxin (TEN; **g**) on relative gene transcription levels of IL-6, IL-8, IL-10 and TNF-α in IL-1ß stimulated HCEC-1CT cells. Cells were pre-incubated with non-toxic concentrations of the respective mycotoxins or 1 µM dexamethasone (Dexa; used as negative control) for 2 h, followed by stimulation with 25 ng/mL IL-1β for an additional 3 h. Gene expression changes are presented as mean + SD of at least three biological replicates, calculated as relative gene transcription (2^−ΔΔCT^), normalized to GAPDH, and compared to the positive control (25 ng/ml IL-1ß). Statistical significances between mycotoxin-treated samples and the positive control were evaluated using the Student’s *t*-test (*p<0.05, **p<0.01, and ***p<0.001). One-way ANOVA followed by a Fisher-LSD post hoc test (a-d; p<0.05) was applied to determine statistically significant differences between concentrations of the same mycotoxin
